# A Novel Method for COVID-19 Diagnosis Using Artificial Intelligence in Chest X-ray Images

**DOI:** 10.3390/healthcare9050522

**Published:** 2021-04-29

**Authors:** Yassir Edrees Almalki, Abdul Qayyum, Muhammad Irfan, Noman Haider, Adam Glowacz, Fahad Mohammed Alshehri, Sharifa K. Alduraibi, Khalaf Alshamrani, Mohammad Abd Alkhalik Basha, Alaa Alduraibi, M. K. Saeed, Saifur Rahman

**Affiliations:** 1 Department of Medicine, Division of Radiology, Medical College, Najran University, Najran 61441, Saudi Arabia; yealmalki@nu.edu.sa; 2 ImViA Laboratory, University of Bourgogne Franche-Comté, 21000 Dijon, France; 3 Electrical Engineering Department, College of Engineering, Najran University Saudi Arabia, Najran 61441, Saudi Arabia; srrahman@nu.edu.sa; 4 Electrical Engineering Department, Victoria University Australia, Sydney 2000, Australia; noman90@ieee.org; 5 Department of Automatic Control and Robotics, Faculty of Electrical Engineering, Automatics, Computer Science and Biomedical Engineering, AGH University of Science and Technology, al. A. Mickiewicza 30, 30-059 Kraków, Poland; adglow@agh.edu.pl; 6 Department of Radiology, College of Medicine, Qassim University, Qassim 51431, Saudi Arabia; fahadalshehri@qumed.edu.sa (F.M.A.); salduraibi@qu.edu.sa (S.K.A.); al.alderaibi@qu.edu.sa (A.A.); 7 Department of Radiological Science, College of Applied Medical Sciences, Najran University, Najran 61441, Saudi Arabia; kaalshamrani@nu.edu.sa (K.A.); mohamedrick@gmail.com (M.K.S.); 8 Faculty of Human Medicine, Zagazig University, Zagazig 44511, Egypt; Mohammad_basha76@yahoo.com

**Keywords:** data analytics, feature extraction, image processing, pandemic, healthcare, chest X-ray images

## Abstract

The Coronavirus disease 2019 (COVID-19) is an infectious disease spreading rapidly and uncontrollably throughout the world. The critical challenge is the rapid detection of Coronavirus infected people. The available techniques being utilized are body-temperature measurement, along with anterior nasal swab analysis. However, taking nasal swabs and lab testing are complex, intrusive, and require many resources. Furthermore, the lack of test kits to meet the exceeding cases is also a major limitation. The current challenge is to develop some technology to non-intrusively detect the suspected Coronavirus patients through Artificial Intelligence (AI) techniques such as deep learning (DL). Another challenge to conduct the research on this area is the difficulty of obtaining the dataset due to a limited number of patients giving their consent to participate in the research study. Looking at the efficacy of AI in healthcare systems, it is a great challenge for the researchers to develop an AI algorithm that can help health professionals and government officials automatically identify and isolate people with Coronavirus symptoms. Hence, this paper proposes a novel method CoVIRNet (COVID Inception-ResNet model), which utilizes the chest X-rays to diagnose the COVID-19 patients automatically. The proposed algorithm has different inception residual blocks that cater to information by using different depths feature maps at different scales, with the various layers. The features are concatenated at each proposed classification block, using the average-pooling layer, and concatenated features are passed to the fully connected layer. The efficient proposed deep-learning blocks used different regularization techniques to minimize the overfitting due to the small COVID-19 dataset. The multiscale features are extracted at different levels of the proposed deep-learning model and then embedded into various machine-learning models to validate the combination of deep-learning and machine-learning models. The proposed CoVIR-Net model achieved 95.7% accuracy, and the CoVIR-Net feature extractor with random-forest classifier produced 97.29% accuracy, which is the highest, as compared to existing state-of-the-art deep-learning methods. The proposed model would be an automatic solution for the assessment and classification of COVID-19. We predict that the proposed method will demonstrate an outstanding performance as compared to the state-of-the-art techniques being used currently.

## 1. Introduction

The Coronavirus has become a pandemic, and the whole world is hugely affected by this pandemic. The Coronavirus is believed to be have first been diagnosed in Wuhan, China [1]. Within no time, the Coronavirus spread across the globe. Cases of the disease it causes have risen rapidly, and health officials have worked to contain the virus. The signs of viral pneumonia were noted in one of the patients on 1 December 2019, in Wuhan, China. This patient is assumed to be the first reported case of COVID-19 and is also cited by a medical report in *The Lancet Journal*. Since then, the chain reaction of reported pneumonia cases started to appear in Wuhan and then throughout the world [1,2,3]. The major symptoms observed in patients were fever, labored breathing, sneezing, and cough. At the end of December, the Chinese government informed the World Health Organization (WHO) of a cluster of pneumonia cases in Wuhan. Between 31 December 2019 and 3 January 2020, 44 cases were reported to the WHO. Wuhan city is connected with other Chinese cities through railway and roads and with other countries through the international airport. The COVID-19 infected people moving from Wuhan to other cities of China and other countries carry the virus. The virus was transmitted when other people were coming in contact with the infected people.

Over the period of a week, the COVID-19 cases appeared across China and Asian countries. As per WHO statistics, 581 confirmed cases of the COVID-19 globally on 23 January 2020. As it was realized that the virus could be transmitted from human to human, the Chinese government lockdown the Wuhan city by disconnecting the local transport and international flights. As of 30 January 2020, with 7818 confirmed cases of the COVID-19, WHO declares COVID-19 as a global public health emergency. Apart from the governments and health officials’ preventive measures, the COVID-19 continues to spread and impact communities globally. The confirmed cases reported till the end of November 2020 were 14 million in the United States of America, 09 million in India, 06 Million in Brazil, and the confirmed cases worldwide reached 65 million as of 30 November 2020. Health officials worldwide are working hard to monitor the COVID-19 and curtail it [4,5,6,7]. The research community is putting its efforts to find technical solutions to automatically detect and isolate the COVID-19 infected people to stop the disease’s spread from human to human.

Artificial Intelligence (AI) is playing a key role in the development of modern tools and technology. AI applications in the healthcare sector are changing and saving lives through quick decision-making, improved image analysis, and priority-based treatment. The variations in the study of a large amount of data in the forms of medical images and laboratory reports could be reduced through AI algorithms which eventually assist the decision support system. Furthermore, the screening and diagnosing process has become easy through AI technology. AI-based mobile applications have been developed to collect and analyze the patients’ data remotely, evaluate the symptoms, and perform self-diagnosis, which eventually reduces physician burnout and saves time and money [8,9,10].

Recently, few studies have been conducted to utilize AI for detecting COVID-19. For example, [11,12] developed a model that can be used for early COVID-19 pneumonia screening. The model can be used via chest computed tomography (CT) images for discriminating between COVID-19, influenza, viral pneumonia, and stable cases utilizing deep-learning techniques. In Reference [13], datasets of disease-infected areas from various nations and 2D and 3D deep-learning techniques for analyzing and detecting suspected COVID-19 patients are implemented. The AI-based analysis was used to obtain high precision in the screening of COVID-19. They also measured the usefulness of the AI algorithm for quantifying and tracking the burden of disease. The deep convolutional neural network model was used for rapid COVID-19 diagnosis with an accuracy of 94.98%. It was concluded that the AI diagnostic algorithm had the advantages of good accuracy, high repeatability, and fast large-scale deployment [14]. A study on AI technologies was performed in tomographic analysis, information analysis, context analysis, machine intelligence, IoT, scientific research of living matter and drugs [15]. The authors discussed the COVID-19 data and how AI can detect the disease early on and treat it. They discussed the role of AI technology in fighting against the emerging COVID-19 pandemic. The comparison was performed using available health data [16] to detect the disease with numerous available technologies, such as Large Data, deep learning, IoT, and AI, in the medical field. For fast and early detection of cases of COVID-19, the AI predictive tools of these technologies have been seen to be useful. They concluded that the AI could assist in COVID-19’s early stage detection. The authors have suggested establishing a foreign data exchange strategy for hospitals and research institutes to deal with the pandemic effectively. For the diagnosis of pneumonia, the chest X-ray image is analyzed by the Generative Adversarial Networks (GAN) and Deep Transfer Learning (DTL) method [17]. Different DTL techniques, such as AlexNet, ResNet18, GoogLeNet, and SqueezNet, have been chosen to diagnose pneumonia from chest X-ray findings. They concluded that ResNet18 is the most acceptable DTL model with a test accuracy of 99%. It has been inferred from the available literature that, although AI has a broad use for healthcare, it is hardly utilized.

Efficient scanning of affected patients, such as separation and care of regular patient scans, is crucial and necessary for resolving harmful Coronavirus. The rRT-PCR [18,19] is currently the key screening technique used to classify COVID-19. The diagnosis is done on the patient’s breathing samples, and results should be available within a few hours to 2 days. The pictorial data from chest X-ray images could be an alternative approach to the PCR screening technique. Many studies such as [20,21] depict the chest imaging of the body could help diagnose COVID-19. Radiologists have also found that patients with symptoms of COVID-19 have CT characteristic imaging features on their lungs, such as peripheral ground-glass opacities and consolidations, which can separate the patient infected with Coronavirus from those diseased with no Coronavirus [22,23]. Researchers conclude that in the diagnosis, measurement, and monitoring of Coronavirus cases, the imaging technique can be an effective tool.

A screening method based on a chest imaging over traditional methods may have many advantages. It may be fast, simultaneously inspect various instances, more accessibility, and utmost significance in clinics with a restrained selection of test tools and facilities. Such a system can be very useful. Moreover, provided the uses of radiology imaging in the advanced healthcare setting, in every hospital, radiology imaging resources are present, making the radiography-based method more accessible and convenient to use.

The research institutes are now trying to counter this pandemic through innovative solutions. Several preprint research articles have been published recently, demonstrating various techniques to detect COVID-19 through chest X-ray images [24,25]. Deep convolutional network was used by [24] to detect COVID-19 positive cases using chest images and achieved the classification accuracy of 91.62 %. Neelima Arora et al. [25] investigated the role of AI in diagnostics of COVID-19. Nevertheless, the performance various models was not investigated for multiclass classification. These methods are encouraging on limited samples of data but are not offering a final solution. Before putting them into practice, these methods will require extensive study and development. As a result, scientists are working to create extremely detailed and efficient AI-based techniques to diagnose and control COVID-19. To identify some special features of COVID-19 patients from chest X-ray images, scientists rely on deep learning (DL) approaches. DL method is very common in different graphical tasks in the recent past, including medical image recognition. By recognizing and correctly classifying patterns in medical images, automatic detection and management of diseases have been revolutionized by DL techniques. The reason for such popularity is that DL techniques do not depend on hand-created elements, but the data themselves automatically acquire characteristics from these algorithms [26]. In the past, DL has helped detect various diseases, using chest X-ray images. CheXNet [27] is a version of a Deep Neural Network (DNN) which detects pneumonia in chest X-ray images. CheXNet has collected outstanding findings, exceeding the cumulative performance of radiologists. A DNN prototype was formulated to detect chest infections using chest X-ray images by a tool named Chest-Net [28].

The evolution of AI-based methods in automated medical analysis and the fast growth in cases of COVID-19 also demanded an automatic AI-based detection and diagnosis device. Several researchers have lately used radiology images to identify COVD-19. Diagnosing COVID-19 using X-ray data of the chest, Hemdan et al. [29] used distinct DL models and suggested a COVIDX-Net network containing seven convolutional neural networks (CNN) models. On a dataset consisting of 224 validated COVID-19 images, Apostolopoulos and Mpesiana [30] have used various pre-trained DL algorithms and obtained 98.75 percent and 93.48 percent precision, respectively, for two and three classes. Livio Fenga [31] developed a statistical analysis tool known as seasonal auto regressive moving average to identify the positivity test rate of the COVID-19. It was reported in [32] that facial recognition using deep learning could be a useful tool for the detection and diagnostics of the COVID infected patients from the crowded places. All these methods are either binary or three-class classifiers. None of the above techniques considers bacterial pneumonia and viral pneumonia as separate groups instead of using COVID-Net.

It has become a great challenge for governments and health organizations to detect suspected Coronavirus patients in crowded places. The available methods are to notice the people’s body temperature and do the lab testing nasal swab samples. The body temperature detection and lab testing of the nasal swabs are complex and intrusive methods. Furthermore, those methods are time-consuming and require a lot of resources. Thus, the contribution of this paper is to develop an AI-based algorithm to detect the suspected Coronavirus patients by classifying the results as normal people, COVID infection, and pneumonia infection. The contributions of this paper based on deep- and machine-learning methods are the following:

1. The proposed deep-learning model consisted of various efficient modules for COVID-19 classification. The inception residual deep-learning model inspires them. We presented different inception residual blocks that cater to information using different depths feature maps at different scales with other layers. The features are concatenated at each proposed classification block, using average pooling and concatenated features to the fully connected layer. The efficient proposed deep-learning blocks used different regularization techniques to minimize the overfitting due to the small COVID-19 dataset.
2. The multiscale features are extracted at different levels of the proposed DL model and embed these features to different ML algorithms to validate the combination of DL and ML models.
3. The RISE-based python-based library was used to visualize the activation of feature maps, and also, the SHAP library was used to check the importance of features extracted from the deep-learning model. The visualization results showed the convergence region receiver operating characteristics (ROC), and precision–recall curves showed our proposed technique performance and validation.

The rest of the paper is organized as follows: A thorough insight into the datasets is given in Section 2. In Section 3, the theoretical framework of the proposed scheme is addressed. Section 4 describes the results and analysis. Finally, Section 4 provides the conclusions of the research.

## 2. Method

The proposed DL model was incorporated in Keras library-backed TensorFlow. The pre-trained models trained on the ImageNet data are used as fine-tuned models. The scikit-learn library is used for the implementation of classical ML algorithms. The adam optimizer with a 1e-4 learning rate was used to optimize the proposed model parameters during training. The cross-entropy loss function was used to calculate the loss between predicted and ground truth labels. The proposed model was run on google collab with 12 GB GPU memory.

### 2.1. Data Collection

**Figure 1:**
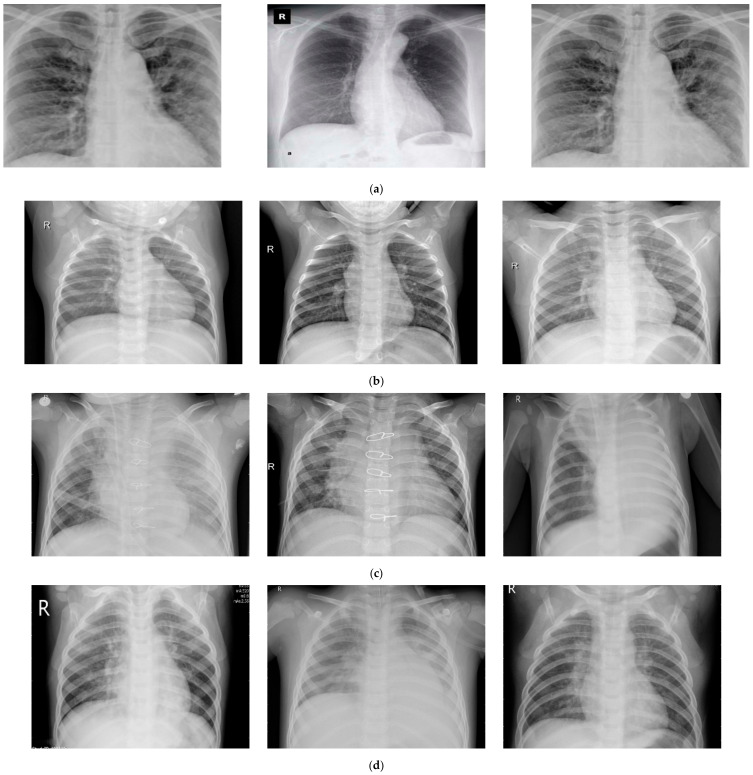
Sample of the collected dataset: (**a**) normal images, (**b**) bacterial pneumonia, (**c**) viral pneumonia, and (**d**) Coronavirus-infection images.

The deep-learning techniques are effective for huge datasets. The physical collection of the COVID-19 data from the laboratories has huge restrictions. Hospitals do not allow for the collecting or sharing of the data, creating a major hurdle in applying DL methods to diagnose and classify COVID-19 patients. The datasets for COVID-19 are available at the open-source database, available Github repository [33]. This study’s data were taken from the Radiological Society of North America (RSNA) and Radiopaedia, etc. This repository contains chest X-rays and CT scan images. This repository is updated regularly, and many researchers have utilized the data from this repository for their research. This repository contains 290 images of chest x-ray images related to COVID-19 patients. The datasets related to pneumonia were collected from the Kaggle repository “Chest X-Ray Images (Pneumonia)” [34]. This dataset contains 1203 normal images, 660 bacterial pneumonia infections, and 931 viral pneumonia infections. A total of 1251 images were taken from these repositories. The benchmark paper has used 1251 images, and due to the same reason, we have used 1251 images in this study. The collected images were resized to 224 × 224 pixels with a resolution of 72 dpi. The dataset details are given in Table 1 and Figure 1. The authors of this paper have taken approval from the ethical committee at the deanship of scientific research at Najran University, Saudi Arabia, for this research.

### 2.2. Development of System Architecture: For Discussion

In this paper, we propose two approaches. In the first approach, the deep-learning models are used for classification and assessment of COVID-19, and in the second approach, the features are extracted from multiple layers of the proposed deep-learning model and passed to various machine-learning models for assessment and multiclass classification, using COVID-19 dataset.

#### 2.2.1. Contribution Using First Approach

In the original Inception-ResNet-v2 model, the author repeated these blocks to provide a deeper model. In our proposed Inception-ResNet blocks, the single inception module was used due to the smaller COVID-19 dataset.

We have proposed Inception-ResNet block A with an extra number of layers branches that consisted of the convolutional layer, using reduction factorization approach. The batch normalization layer was used after each 1 × 1 Conv, 3 × 3 Conv, 5 × 5 Conv layers. The number of filters in each convolutional layer branch keeps the same as proposed in the original Inception-ResNet-v2.

#### 2.2.2. Contribution Using the Second Approach

**Figure 2:**
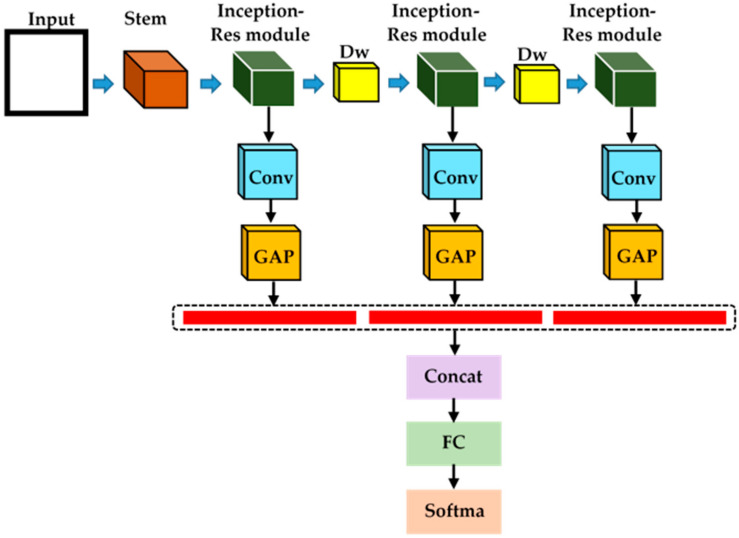
The proposed model is based on the Inception-ResNet module with multiscale feature extraction and concatenation for COVID-19 classification.

For the second contribution, the features are extracted from a different layer of the proposed model and concatenate these features using the average-pooling layer. After concatenation, the feature vector passed to the fully connected and classification layer is shown in Figure 2. The feature extraction from multiple levels in the proposed model produced a better performance than simply fine-tuned the deep-learning models.

Contribution using the second approach:

- The objective of the second approach is to combine deep-learning and machine-learning models.
- The features are extracted from the proposed deep-learning model and passed features to the traditional machine-learning model for classification of COVID-19.
- The multiscale features are extracted from various blocks from proposed Inception-ResNet blocks are concatenated, and after the concatenation of these features are used in machine-learning classifiers.

The feature extraction at a different level of pre-trained models and fusion of these multiscale features extracted from different levels of deep-learning model gives better performance, especially with traditional deep-learning models.

#### 2.2.3. Approach 1: Proposed CoVIRNet Deep Learning

The various deep-learning models were used for classification detection and segmentation, using the COVID-19 dataset. The model is designed based on different features from proposed architectures and concatenated at the final fully connected layer. The block diagram is shown in Figure 2. We named our proposed model CoVIRNet (COVID Inception-ResNet model).

Kaiming He et al. in [35] presented residual connection in the ResNet model and widely used in image classification and object recognition. The authors proved that these residual connections could be used to train the deeper neural network. The author claimed that deep residual connections always do not provide better training for deeper neural networks. The additional measurement needs to take to improve the efficiency of deeper networks. The inception of deep-learning GooLeNet or Inception-v1 was proposed in and later the inception-based architectures in various forms. Szegedy et al. [36] has introduced series of inception-based models, such as Inception-v2, Inception-v3, Inception-ResNet-v1, and Inception-ResNet-v2. The detail of each inception-based model can be found [36]. The proposed model for COVID-19 classification is based on inception.

ResNet-v2 architecture. The inception of ResNet-v2 architecture consisted of various residual-based blocks. Inception-ResNet blocks A, B, and C were introduced in Inception-ResNet-v2 with some reduction blocks—A and B. The mean idea behind the proposed inception-based resentment block is to capture spatial information with various positions and scales using a parallel branch with different filter sizes such as 1 × 1, 3 × 3, 5 × 5, 7 × 7. The bigger and smaller filter size captured the different receptive fields from the input image with different scales. In the original Inception-ResNet-v2 model, the author repeated these blocks to provide a deeper model. In our proposed Inception-ResNet blocks, the single inception module was used due to the smaller COVID-19 dataset.

**Figure 3:**
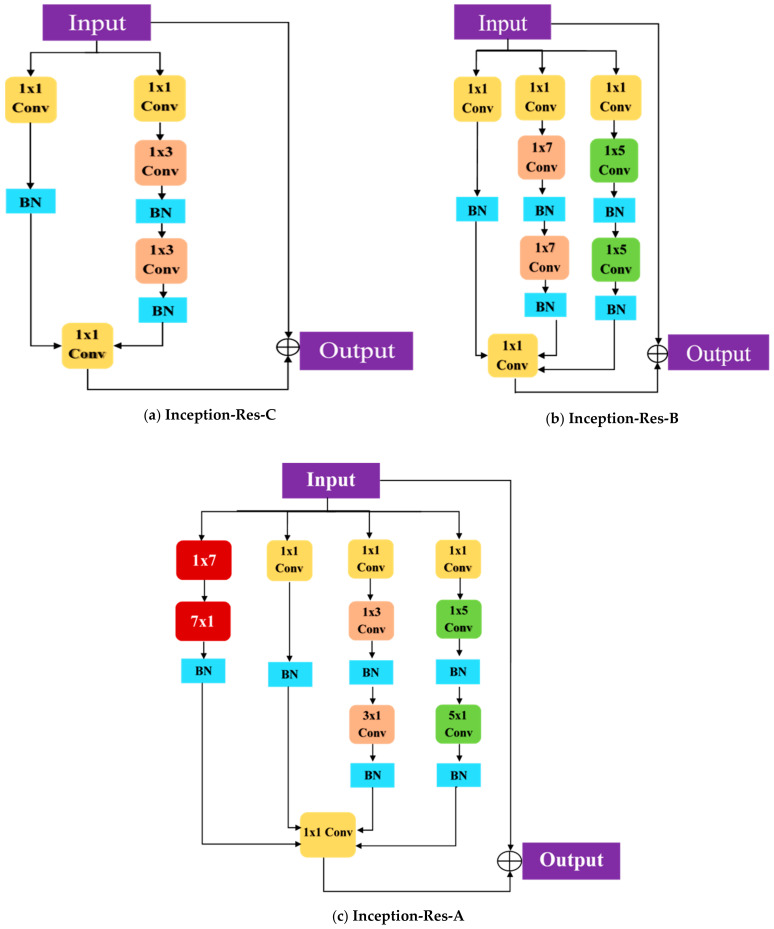
The Inception-ResNet blocks used in our proposed model.

Our proposed Inception-ResNet blocks A, B, and C is shown in Figure 3. We have proposed Inception-ResNet block A with an extra number of layers branches consisting of convolutional layers using the reduction factorization approach, as shown in Figure 3. The batch normalization layer was used after each 1 × 1 Conv,3 × 3 Conv, and 5 × 5 Conv layers. The number of filters in each convolutional layer keeps the same as proposed in the original Inception-ResNet-v2. The second contribution is that the features are extracted from a different layer of the proposed model and concatenate these features by using the average-pooling layer. After concatenation, the feature vector passed to the fully connected and classification layer, as shown in Figure 2. The feature extraction from multiple levels in the proposed model gives better performance than simply fine-tuned the deep-learning models as shown in Table 2.

**Figure 4:**
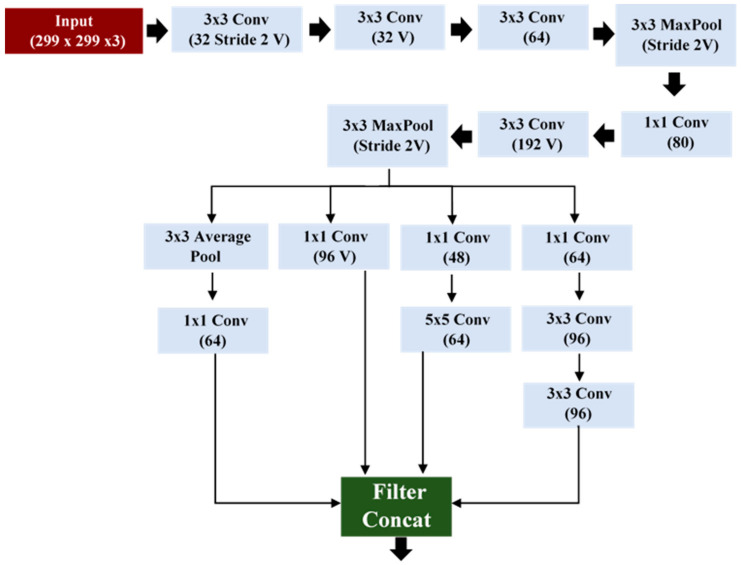
Stem block used in the proposed model.

The stem block used in our proposed model is shown in Figure 4. The stem block used various layers with different feature maps and concatenated them to get spatial information at different depths. The proposed model is used for the extraction of features from different layers with different depths and gives useful semantic features information for COVID classification.

#### 2.2.4. Approach 2: Deep-Feature-Extraction and Machine-Learning Models

**Figure 5:**
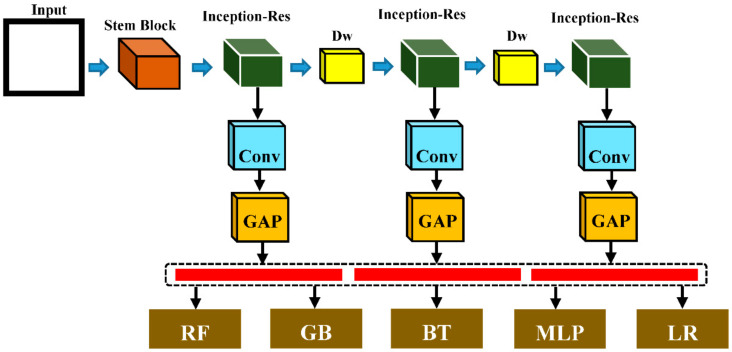
The proposed deep-learning model used as feature extraction and various machine-learning classifiers used as a classification of COVID-19.

The second approach for COVID-19 classification is to combine deep- and machine-learning models. The features are extracted from the proposed deep-learning model and passed to the traditional machine-learning model to classify COVID-19. The multiscale features are extracted from various blocks from the proposed Inception-ResNet blocks and concatenated. After the concatenation, these features (red lines show feature extraction from each convolutional layer) are used in machine-learning classifiers. This excellent approach performance in our proposed model and produced excellent results as compared to existing state-of-the-art models. The proposed approach based on multiscale feature extraction with classification is shown in Figure 5.

##### Random Forest

RF [37] is a famous machine-learning model built on various decision tree combinations. The random forest used the random subsets of the bootstrapped dataset and also picked the random subset of features. The random forest created 100–1000 decision trees. The random tree approach has made the random forest a powerful machine-learning algorithm.

##### Bagging Tree

Bagging tree [38] or classifier is a bootstrap aggregating ensemble method that used a training set by resampling and running algorithms. It creates multiple datasets based on the bootstrapped method and used algorithms on each of them. It combined all models obtained from all bootstrapped datasets and took an average of them, using averaged classifier/combined classifier and then predicting the output on the averaged or final classifier. It may overfit due to large samples are repeated and used in training the classifier.

##### Gradient Boosting Classifier

The idea of Boosting [39] is initially used as a technique to elevate the performance of low-performing students into good students by increasing their learning capacity. The working of gradient boosted machine (gbm) can systematically be understood using the AdaBoost algorithm. In the AdaBoost algorithm, each observation is assigned similar weights for the training of the decision tree. Then, the decision trees that are hard to classify are favored with higher weights than the trees that are easier to classify, given lower weights. This weight distribution is based on the difficulty level of classification, and the second tree starts to grow based on the outcomes and predictions from the first tree. Until now, the model stands at the aggregate of the first two trees. At this stage, the errors for classification filters are estimated for this 2-tree ensemble model, and the residuals are here, and then they are further investigated and processed by the third tree. This process is repeated for fixed iterations. This process enables all the upcoming trees to pick up the observations that previous trees missed to identify. Finally, we get the final weighted sum built upon all of the previous tree model estimations. This weighted sum is then the overall process’s outcome, resulting in the final predictions from the last tree ensemble model. On a similar note, the GB algorithm is also a step-wise and integral process that trains the model based on previous trees’ feedback, and the outcomes reflect the filtering from the first to the last tree. The stand-out difference between AdaBoost and GB algorithms is how both of these algorithms classify the limitations and weaknesses of low-performing decision trees. The comparative working difference can be stated so that AdaBoost increases the weights of poor-performing decision trees, while GB does the same but through the gradient in the loss function. The AdaBoost has another limitation in terms of configuring the models according to user or application requirements. The advantage of using GB instead of AdaBoost is that the GB algorithm provides more control to the user by allowing the user to tune the cost function according to the application requirement.

##### Perceptron Model Multilayer

The MLP model is compromised of the input layers, which have source nodes, followed by the computational nodes residing in the hidden layers and the nodes in the output layer. The MLP model has a limitation of training the model in a supervised fashion. If we had a smaller amount of data, it would be difficult to train the MLP based model due to lots of parameters need to trained the MLP model with fewer datasets. The main idea to used MLP for classification problem that’s the reason, we used CNN model with some feature extraction from different layers with classical machine-learning classifiers to tackle the less labeled dataset.

This shortcoming is addressed with the back-propagation algorithm [40], which enables supervised learning for MLP. The back-propagation algorithm works in two steps with the help of two types of networks. The forward pass network is used to estimate the outputs according to the inputs, while the backward pass network is used to feedback the partial derivatives of the defined cost function.

##### Logistic Regression

The LR algorithm [41] is used for classification problems that work in a supervised learning fashion. The LR model is primarily used to estimate the likelihood probability of the target variable. The possibility or an outcome of the presence test for a target variable is binary, resulting in either the true or false result. Typically, LR is also referred to in the literature as binary LR aimed for the classification of binary target variables. However, it can also be extended based on required categories to predict the likelihood of more than two types of target variables.

## 3. Results and Discussions

The different deep-learning pre-trained models, such as Xception, ResNet101, DensNet201, and MobielNetV2, are fine-tuned, and only trained weights of the last few layers are used. The comparison of the performance of these deep-learning models with the proposed model is shown in Table 2. The performance measures such as accuracy, precision, recall, F1score, and visualization tools such as ROC shows better performance of our proposed model, and we can see the effectiveness of our proposed model with state-of-the-art methods. The proposed model produced a better performance than fine-tuned models, and the proposed model with machine-learning random-forest classifiers achieved excellent performance, as shown in Table 2.

**Figure 6:**
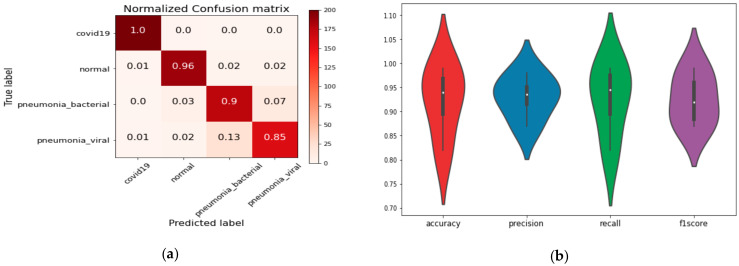

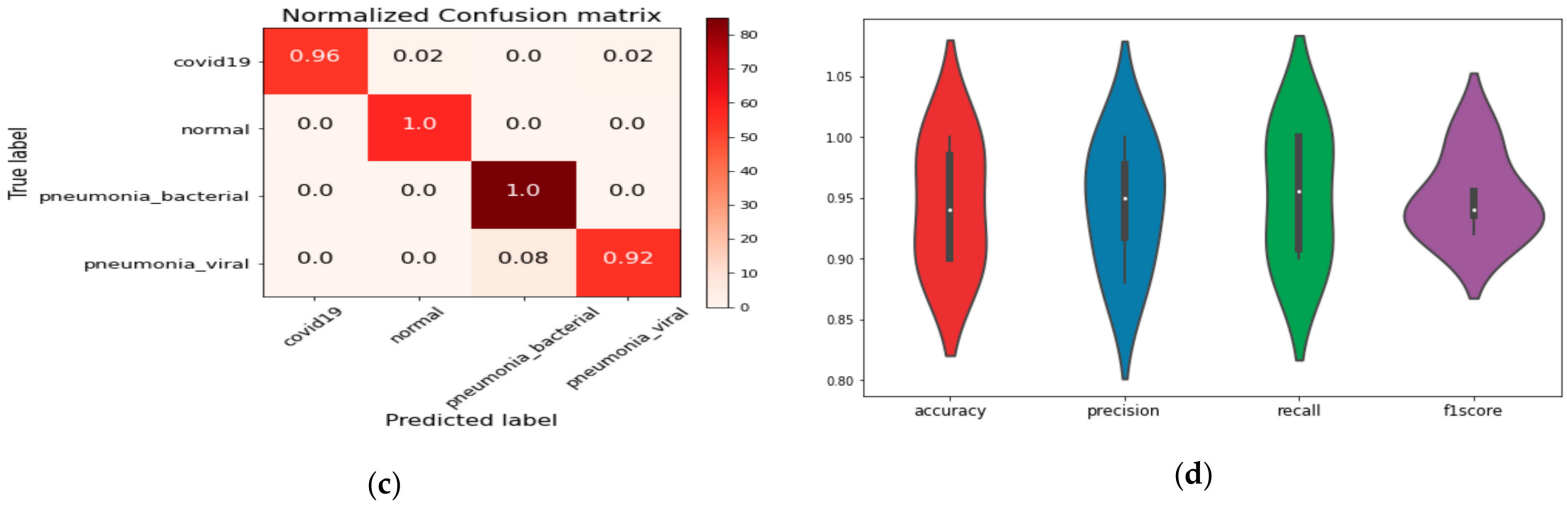
(**a**) The confusion matrix for Xception model. (**b**) Performance analysis based on Xception DL model. (**c**) Confusion matrix for CoVIRNet DL model. (**d**) Performance analysis based on CoVIRNet DL model.

The confusion matrix and performance analysis based on overall accuracy, precision, recall, and F1score for all classes, using the proposed model and Xception model, are shown in Figure 6. We showed only the performance comparison based on the confusion matrix and performance analysis metrics based on best and bad deep-learning models.

**Figure 7:**
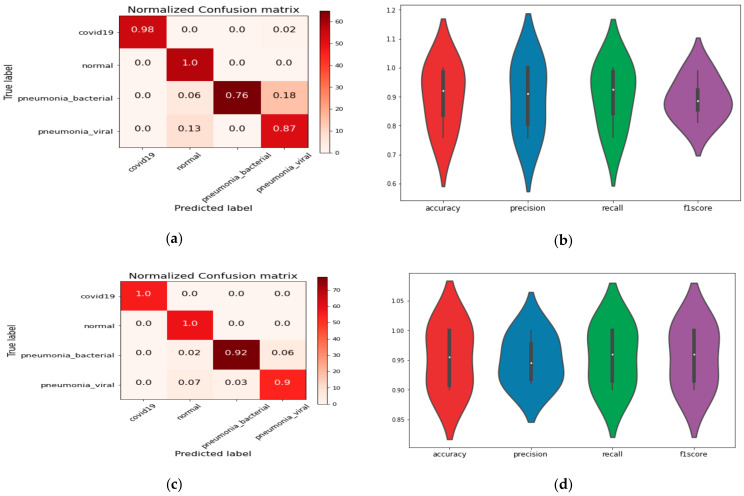
(**a**) The confusion matrix and performance analysis of all classes based on a proposed model with logistic regression (LR) model and (**b**) proposed CoVIRNet model with random forest (RF). (**c**) Performance metrics using logistic regression and (**d**) proposed CoVIRNet model with random forest.

Similarly, the performance analysis metrics and confusion matrix for the proposed model with logistic regression and the random-forest machine-learning model are shown in Figure 7. The proposed model with random forest achieved good performance for all classes, as shown in the confusion matrix in Figure 7c.

**Figure 8:**
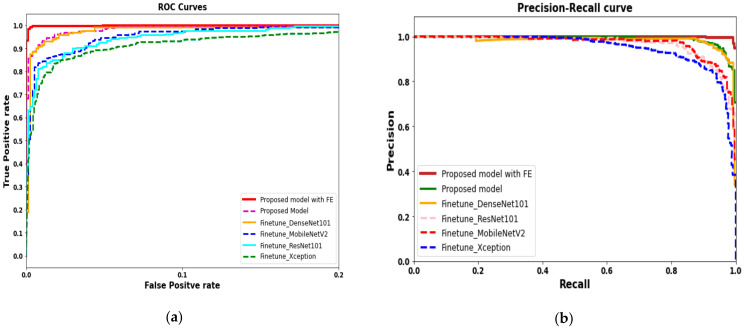
(**a**) ROC for proposed deep-learning model with and without feature extraction compared with an existing deep-learning model. (**b**) Precision–recall curves for proposed model compared with existing state-of-the-art deep-learning models.

The region of convergence and precision–recall curves is further used to analyze and assess the proposed model with fin tuned deep-learning models is shown in Figure 8. The proposed model generated ROC (red line in Figure 8a) curve showed significant performance improvements compared to fine-tuned deep-learning models. The precision–recall curve based on the proposed model also showed a significant difference compared to PR curves generated by fine-tuned models shown in Figure 8b.

The performance metrics, accuracy, precision, recall, and F1score, for the proposed model with random forest and other traditional machine-learning classifier are shown in Table 3. The proposed model using random forest classifiers achieved excellent performance compared to different classifiers, as shown in Table 3.

**Figure 9:**
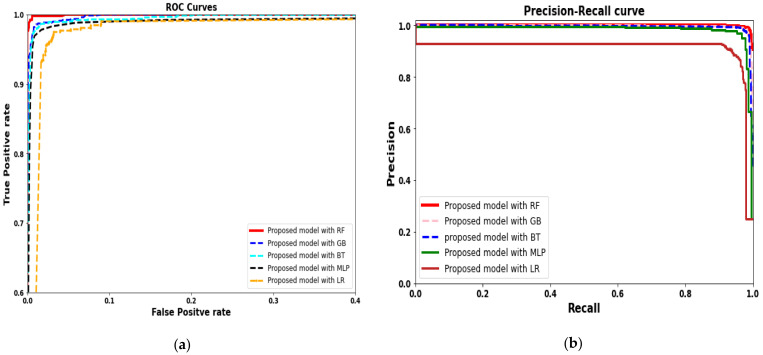
(**a**) ROC for proposed deep-learning model, with feature extraction using various machine-learning models. (**b**) Precision–recall curves for the proposed model, with feature extraction using various machine-learning models.

The ROC and PR curves based on the proposed model using different classifiers are shown in Figure 9. The ROC curves generated by proposes model with random forest achieved good performance as compared with other classifiers. The LR machine-learning model with the proposed deep-learning model achieved a lower performance curve that further validates our LR results shown in Table 3. Similarly, the PR curves also showed a similar pattern for the proposed model with random forest and other classifiers.

Lundberg and Lee (2016) [42] proposed SHAP (Shapley Additive exPlanations) is a technique that is widely used to explain individual predictions. It is based on the game theory approach and could explain the importance of features in the proposed model. The deep-learning internal structure is just like a black-box and sharply feature importance well explain the explainable of deep-learning models. The idea behind SHAP feature importance is simple, and features with large absolute Shapley values are important.

**Figure 10:**
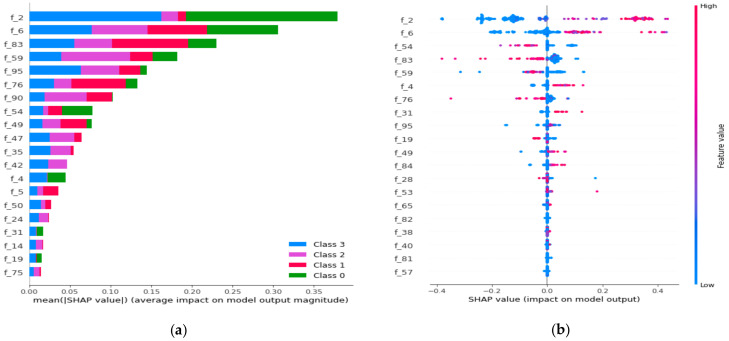
(**a**) The proposed model’s feature importance, using the random forest for four classes; (**b**) the feature importance impact, using SHAP library, shows high and low importance.

The SHAP python-based library was used to further validate our proposed model’s results with machine-learning classifiers is shown in Figure 10. Figure 10a shows the distribution of feature importance extracted from the proposed model using a random forest algorithm. Figure 10b also validated the feature importance and showed that f_2 and f_6 have the highest feature importance values. In our case, we extracted 512 full features from the last fully connected layer of the proposed model. We have named these features f_1, f_2, and so on, from start to end (total 512), as shown in Figure 10. The earlier features, we can say f_2 and f_6 are the most important features as compared to other features. We have used these features in the traditional machine-learning models to get a better score as compared to all features that are extracted from the last fully connected layer from proposed deep-learning models. Based on the experimental verification, we have used the first three hundred (300) features out of five hundred and twelve (512) for the machine-learning model and improved the performance, as shown in the last row of Table 4. The SHAP python-based library could help extract useful or important features for every class in the trained dataset that will be useful for classification decision-making.

The summary plot combines feature importance with feature effects. Each point on the summary plot is a Shapley value for a feature and an instance. The position on the *y*-axis is determined by the feature and on the *x*-axis by the Shapley value. The color represents the value of the feature from low to high. Overlapping points are jittered in the *y*-axis direction, so we get a sense of distributing the Shapley values per feature. The features are ordered according to their importance.

### 3.1. Data Visualization

Deep neural networks are being used increasingly to automate data analysis and decision-making. However, their decision-making process is largely unclear and is difficult to explain to the end-users.

We have used RISE, which generates an importance map indicating how salient each pixel is for the model’s prediction. In contrast to white-box approaches that estimate pixel importance using gradients or other internal network states, RISE works on black-box models. It estimates importance empirically by probing the model with randomly masked versions of the input image and obtaining the corresponding outputs. This approach is general and applies to any off-the-shelf image network, treating it as a complete black box and not assuming access to its parameters, features, or gradients. The key idea is to probe the base model by sub-sampling the input image via random masks and recording its response to each of the masked images. The final importance map is generated as a linear combination of the random binary masks. The combination weights come from the output probabilities predicted by the base model on the masked images. This seemingly simple yet surprisingly powerful approach allows us to peek inside an arbitrary network without accessing any internal structure. Thus, RISE is a true black-box explanation approach that is conceptually different from mainstream white-box saliency approaches such as GradCAM and, in principle, is generalizable to base models of any architecture.

The visualizations are an important part of the statistical model analysis. They facilitate in understanding the working of any learning-based model. For instance, the use of the RISE library in python helps to interpret the qualitative type results for the sample input variables of a model. We have extracted features from the last fully connected layer from proposed deep-learning models and proposed explainability. The RISE python-based library could be useful for the feature interpretation and judge the deep-learning models’ explainability. The RISE library has some interpretation to visualize the activation of the trained weights and gradient of the last layer in our proposed model.

We have adopted the RISE and generated the saliency map from the predictions of the last layer of the proposed model. From the given X-ray images, we have randomly created 1000 masked variants and utilized these masks to get the activation map of the gradient of the trained model.

**Figure 11:**
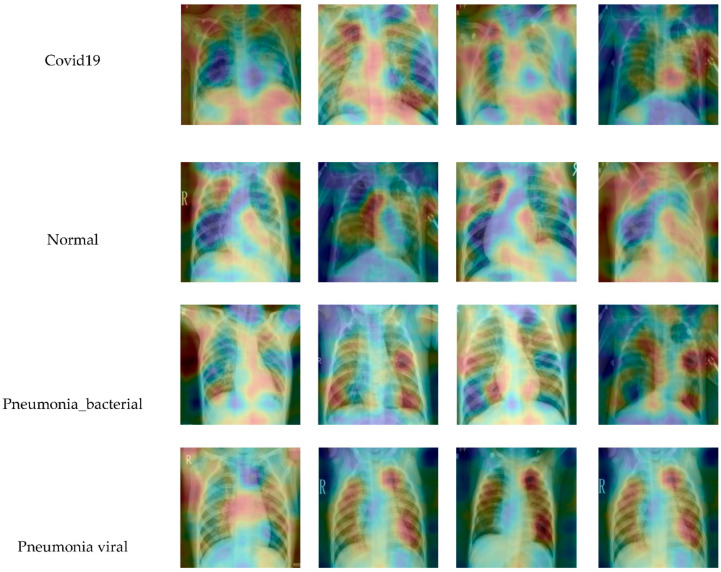
The visualization of some predicted samples based on our proposed model, using the RISE library.

The blue color at some test X-ray sample images in Figure 11 shows more activation of gradient-based trained weights of the last layer of the proposed classification model for all classes. We used the weights and gradient of the last layer of our proposed model to visualize the saliency map.

This color pattern visualization of the proposed model could better understand the weights activation used for model overfitting interpretation and could be helpful for clinical applications. The attention regions correspond to the right features from a radiologist’s perspective shown in the activation map for few samples in Figure 11. The notion behind the use of the RISE visualization tool is to achieve better and improved classification scores from the generated image’s semantical sections. This also enabled the final output mask for each class to yield higher weights. Figure 11 presents the feature map importance and shows the corresponding right location of features. It would be better to see the feature maps activation of the respective class where the features provide more attention for the good features. The red and green color shows the most important features in a different region of the predicted model. It shows the effectiveness of the proposed model in an explainable way. Some regions at outlier in the X-ray image boundary did not correspond to the proposed model’s actual prediction as shown in Figure 11 and produced a small false positive number of pixels.

**Figure 12:**
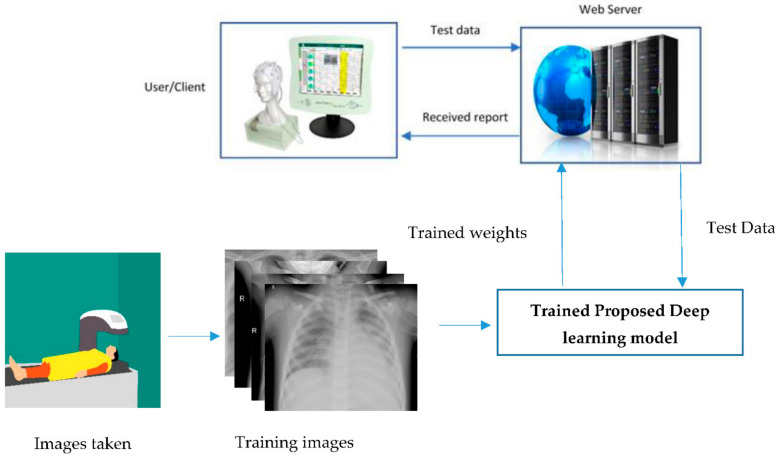
Deployed proposed deep-learning model for assessment and detection of COVID-19 in clinical application.

The proposed model based on deep learning is implemented for clinical application and could be used to diagnose COVID-19 detection. The proposed deep-learning models are trained. The weights of trained models are saved on the internet and need to deploy training models in clinical applications. The following numbered steps were already completed in this research study: (1) load saved the weight of the proposed model, (2) preprocess testing dataset, and (3) perform the actual prediction on testing dataset and handle very well the prediction response based on testing dataset. Now the further steps need to be taken to get accurate results on the test dataset remotely. A trained model with weights is loaded, and all dependencies related to model training are included in the web server for further prediction of the user outcome for clinical application. The proposed model trained on the training dataset can be deployed in hospitals using a web server remotely. The testing X-ray images obtained from the patients will be sent to the webserver that is deployed in the hospital using either cloud or local server. The trained model that is used for prediction was deployed in a web server, and based on the testing patient dataset, the model will predict either healthy and some infection condition of patients and will send back the report to client or user. The complete process is shown in Figure 12. We deployed our trained model by using Flask, which is a lightweight web framework. It is easy to use for the basic web application used on various platforms and easy to send your model in a production written based on python language.

### 3.2. Discussion

In this paper, the efficient deep-learning model was proposed to classify four class COVID-19 images using famous machine-learning algorithms, such as random forest, bagging tree, gradient boosting, MLP, and logistic regression. The multiscale or multilevel features are used in our proposed model for the classification of COVID-19 images. The multiscale features provide useful high-level and low-level semantic information from input feature maps at various feature maps depth, location, and various scales. The multiscale or multilevel features are used in our proposed model for the classification of COVID-19 images. The multiscale features provided useful high-level and low-level semantic information from input feature maps at various feature maps depths, locations, and scales.

Furthermore, the multiscale features extracted from various proposed deep-learning models were used in machine-learning algorithms without using any handcrafted feature extraction approach. The deep-learning models trained on COVID-19 images and produced efficient automatic features to discriminate feature space for each class in the COVID-19 dataset. The main influence of the deep-learning features formed by the proposed deep model is ascribed to the fact that these machine-learning algorithms learned the utmost discriminant parameters for the classification of COVID-19 images. The proposed deep-learning model produced features that would be separated well of each class and produced excellent results on the COVID-19 dataset.

The state-of-the-art deep-learning models used as fine-tuned (trained some layers) also produced a better performance. Especially DenseNet201 pre-trained models produced the performance that is very close to our proposed deep-learning model. We also observed that our proposed model with random forest achieved the highest performance amongst existing deep-learning models. The performance analysis of existing deep-learning models with our proposed model with feature extraction and classification is shown in Table 4. The different authors used different datasets, and different parameters for the training and optimization of their proposed techniques are shown in Table 4. However, we compared our proposed model’s performance with the existing model that used the same dataset samples with the same number of classes. Our proposed model produced excellent performance compared to other deep-learning models on the same dataset [43,44,45,46,47,48,49,50,51]. Wang and Wong [47] proposed a deep-learning model using the same dataset used in our research and gained good classification accuracy of 83.5% for four classes. Khan et al. [51] achieved 89% classification accuracy. We have used the same dataset as used by Khan et al. [51] and shown the comparison of our proposed model with the base paper (Khan et al. [51]) in Table 4. The Khan et al. [51] proposed simple Xception architecture pre-trained on the ImageNet dataset and compared the results with another pre-trained model. We have proposed two scenarios: In the first method, we proposed a deep-learning Inception-ResNet block with fewer parameters for smaller datasets and extract features from different layers of the proposed model. Furthermore, we fused these features from different layers with different scales, depths, and different feature maps and, after fusion, pass these features to deep-learning classifiers, such as softmax, and also compared a bunch of pre-trained deep-learning models with our proposed model. In the second approach, the automatic deep feature extraction and fusion done in the first phase are passed to the classical machine-learning methods for COVID-19 assessment.

Recently, Tanvir Mahmud et al. [52] used the Stacked Multi-Resolution CovXNet deep-learning model and achieved 90.3% classification accuracy, using four class datasets. Our proposed CoVIRNet model produced excellent performance is shown in bold in Table 4.

The huge number of COVID-19 patients has increased the burden on the healthcare systems. The traditional RT-PCR test kits are limited in quantity, and it is quite difficult to diagnose the patient with respiratory issues through these test kits. Furthermore, this testing of suspected patients through these test kits is time-consuming. The diagnostics of the suspected COVID patients through chest X-rays could save time and resources as X-ray devices are still present in most hospitals. Thus, this research paper proposes an AI-based chest X-ray data analysis technique for the initial diagnostics of the COVID infection and prioritizing patients’ options for further RT-PCR. The proposed technique would be beneficial in an inpatient environment for the segregation of COVID patients. The proposed CovidAID: COVID-19 AI Detector can automatically detect and diagnose the COVID-19 patients. It would be useful in hospitals where expert radiologists are not available to analyze the X-ray data. The performance of the proposed method in this paper has been compared with other published papers [43,44,45,46,47,48,49,50,51,52] and has been shown in the Table 4.

## 4. Conclusions

The COVID-19 pandemic cases are rising daily. There is a shortage of resources in many countries, and it is critical to identify every suspected positive infection as quickly as possible to stop the spread of the infection. This paper presented some initial findings using a DL model to detect COVID-19 positive cases from chest X-ray images. Important performance improvements were demonstrated on the same chest X-ray-pneumonia dataset over the COVID-Net dataset. The findings look positive, although the size of the dataset that is publicly accessible is limited. The proposed approach is based on the CNN model designed to use chest X-ray images to classify COVID-19 infections. The performance of the proposed technique was verified on a small publicly accessible chest X-ray images dataset of different pneumonia and COVID-19 cases. The proposed CoVIRNet is cost-effective in terms of measurement and produces promising results. We predict that the proposed CoVIRNet will be a useful tool for radiologists and health officials to gain a better understanding of important aspects associated with COVID-19 cases.

## Figures and Tables

**Table 1:** Dataset details.

Sr. No.	Disease	Total Samples	Training Samples	Testing Samples
1	Normal images	310	248	62
2	Pneumonia-bacterial-infection images	330	264	66
3	Viral-pneumonia images	327	261	66
4	Corona-infection images	284	227	57

**Table 2:** Performance analysis of deep-learning models on COVID-19 for four classes.

Model	Accuracy	Precision	Recall	F1score
Xception—Fine-Tuned	0.8822	0.88041	0.8796	0.8787
CoVIRNet Model	0.9578	0.9491	0.9544	0.9509
ResNet101—Fine-Tuned	0.8880	0.8954	0.9033	0.8923
MobielNetV2—Fine-Tuned	0.90347	0.9028	0.9011	0.9005
DenseNet201—Fine-Tuned	0.9419	0.9462	0.9514	0.9474
CoVIRNet with RF	0.9729	0.9774	0.9702	0.9732

**Table 3:** Performance analysis of deep-learning and machine-learning model. Feature extracted from deep-learning proposed model and classify with machine-learning classifiers.

Model	Accuracy	Precision	Recall	F1score
CoVIRNet Model with LR	0.9279	0.9283	0.9264	0.9263
CoVIRNet Model with MLP	0.9446	0.9439	0.9437	0.9435
CoVIRNet Model with GB	0.9523	0.95141	0.9515	0.9512
CoVIRNet Model with BT	0.9613	0.9607	0.9607	0.9605
CoVIRNet Model with RF	0.9729	0.9774	0.9702	0.9732

LR, logistic regression; MLP, multilayer perceptron layer; GB, gradient boosting model; BT, bagging tree; RF, random forest.

**Table 4:** The comparison study for COVID-19 classification using deep and ML models.

Study	Dataset	Model Used	Classification Accuracy
Narin et al. [43]	2-class: 50 COVID-19/50 normal	Transfer learning with ResNet50 and Inception-v3	98%
Panwar et al. [44]	2-class: 142 COVID-19/ 142 normal	nCOVnet CNN	88%
Altan et al. [45]	3-class: 219 COVID-19 1341 norma l1345 pneumonia viral	2D curvelet transform, chaotic salp swarm algorithm (CSSA), EfficientNet-B0	99%
Chowdhury et al. [46]	3-class: 423 COVID-19 1579 normal 1485 pneumonia viral	Transfer learning with CheXNet	97.7%
Wang and Wong [47]	3-class: 358 COVID-19/5538 normal/8066 pneumonia	COVID-Net	93.3%
Kumar et al. [48]	3-class: 62 COVID-19/1341 normal/1345 pneumonia	ResNet1523 features and XGBoost classifier	90%
Sethy and Behera [49]	3-class: 127 COVID-19/127 normal/127 pneumonia	ResNet50 features and SVM	95.33%
Ozturk et al. [50]	3-class: 125 COVID-19/500 normal 500 pneumonia	DarkCovidNet CNN	87.2%
Khan et al. [51]	4-class: 284 COVID-19/310 normal/330 pneumonia bacterial/327 pneumonia viral	CoroNet CNN	89.6%
Tanvir Mahmud et al. [52]	4-class: 305 COVID-19 + 305 Normal + 305 Viral Pneumonia + 305 Bacterial Pneumonia	StackedMulti-resolutionCovXNet	90.3%
Proposed CoVIRNet DL model	4-class: 284 COVID-19/310 normal/330 pneumonia bacterial/327 pneumonia viral	Multiscale features CoVIRNet	95.78%
Proposed CoVIRNet DL model with RF	4-class: 284 COVID-19/310 normal/330 pneumonia bacterial/327 pneumonia viral	Multiscale features CoVIRNet+ RF	97.29%

## Data Availability

Not Applicable.
